# Supplementary material for: P-TRAP: a Panicle Trait Phenotyping tool
Source: BMC Plant Biol. 2013 Aug 29;13:122. doi: 10.1186/1471-2229-13-122 (PMC3848748; doi:10.1186/1471-2229-13-122)
Supplement: Additional file 7 — P-TRAP source code. The Netbeans project that contains the source code of the application. [file 1471-2229-13-122-S7.zip › javadoc.zip!/javadoc/org-bioalg-widgetfactory/org/bioalg/widgetfactory/package-tree.html]

org.bioalg.widgetfactory Class Hierarchy (WidgetFacory)


JavaScript is disabled on your browser.


- Package
- Class
- Tree
- Deprecated
- Index
- Help

*org.bioalg.widgetfactory 1.0*

- Prev
- Next

- Frames
- No Frames

- All Classes

# Hierarchy For Package org.bioalg.widgetfactory

## Class Hierarchy

- java.lang.Object
  - org.netbeans.api.visual.widget.Widget (implements javax.accessibility.Accessible, org.openide.util.Lookup.Provider)
    - org.bioalg.widgetfactory.CircleWidget
    - org.bioalg.widgetfactory.ParticleWidget
  - org.bioalg.widgetfactory.WidgetPopUpMenuHandler (implements java.awt.event.ActionListener, org.netbeans.api.visual.action.PopupMenuProvider)

## Enum Hierarchy

- java.lang.Object
  - java.lang.Enum<E> (implements java.lang.Comparable<T>, java.io.Serializable)
    - org.bioalg.widgetfactory.WidgetPopUpMenuHandler.WIDGET\_COMMANDS

- Package
- Class
- Tree
- Deprecated
- Index
- Help

*org.bioalg.widgetfactory 1.0*

- Prev
- Next

- Frames
- No Frames

- All Classes
